# Characterization of the physical properties of tumor-derived spheroids reveals critical insights for pre-clinical studies

**DOI:** 10.1038/s41598-019-43090-0

**Published:** 2019-04-29

**Authors:** Ludivine Guillaume, Lise Rigal, Jérôme Fehrenbach, Childérick Severac, Bernard Ducommun, Valérie Lobjois

**Affiliations:** 1 ITAV, CNRS, UT3, Université de Toulouse, Toulouse, France; 2 IMT, CNRS, UT3, Université de Toulouse, Toulouse, France; 3 CHU de Toulouse, Toulouse, France

**Keywords:** Cancer models, Tissue engineering

## Abstract

Three-dimensional spheroids are widely used as cancer models to study tumor cell proliferation and to evaluate new anticancer drugs. Growth-induced stress (i.e., stress that persists in tumors after external loads removal) influences tumor growth and resistance to treatment. However, it is not clear whether spheroids recapitulate the tumor physical properties. Here, we demonstrated experimentally and with the support of mathematical models that, like tumors, spheroids accumulate growth-induced stress. Moreover, we found that this stress is lower in spheroids made of 5,000 cancer cells and grown for 2 days than in spheroids made of 500 cancer cells and grown for 6 days. These two culture conditions associated with different growth-induced stress levels also had different effects on the spheroid shape (using light sheet microscopy) and surface topography and stiffness (using scanning electron microscopy and atomic force microscopy). Finally, the response to irinotecan was different in the two spheroid types. Taken together, our findings bring new insights into the relationship between the spheroid physical properties and their resistance to antitumor treatment that should be taken into account by the experimenters when assessing new therapeutic agents using *in vitro* 3D models or when comparing studies from different laboratories.

## Introduction

All tissues are characterized by physical parameters to which cells respond and adapt. Tissue mechanics studies have been developed to understand their stress and strain responses. In the 1990s, Fung and collaborators demonstrated the fundamental importance of residual stress, which corresponds to stress remaining in a tissue when all external loads have been removed^1^. Making cuts and observing the resulting changes of shape can reveal the presence of residual stresses in a tissue *ex vivo*. By using this experimental method, the presence of residual stresses has been demonstrated in arterial wall, veins, heart and brain, for instance^2–5^. An important conclusion of these studies is that the level of internal stress and strain of organs guides physiological functions.

Tumor micro-regions consist of heterogeneous cancer cell populations that are organized in 3D and in which cell growth and response to anti-tumor drugs are strongly influenced by the 3D architecture, cell-cell junctions and interaction with the microenvironment^6,7^. Moreover, tumor growth and progression are also strongly influenced by mechanical cues^6,7^. In solid tumors, cancer cells proliferation imposes elastic strain on the surrounding tumor microenvironment, thus generating a stress created by reciprocal forces between solid tumor and the surrounding normal tissue. Stylianopoulos *et al*.^8^ have experimentally demonstrated the presence of residual stresses in murine and human solid tumors. Indeed, partial cut through an excised tumor causes opening due to relaxation of the growth-induced stress in the absence of external loads.

Cells cultured in 2D monolayers have been classically used for the pharmacological evaluation of anticancer drugs. However, over the last decade, the development of 3D cell culture models has significantly changed the landscape of preclinical studies, and 3D spheroid models are now widely used as tumor surrogates. Indeed, spheroids in which cancer cells are cultured as 3D aggregates reproduce the cell-cell and cell-matrix interactions found in solid tumors^9^. Moreover, spheroids can grow up to several hundred micrometers in diameter, thus progressively displaying a gradient of proliferating cells similar to what observed in tumor micro-regions^10^. In recent years, technical innovations in the development of dedicated devices and strategies to produce and manipulate spheroids and the marketing of ready-to-use spheroids have tremendously boosted the use of such models for basic research, drug screening, and preclinical studies^11–13^. In parallel, many efforts have been made to develop new technologies to characterize the complex 3D multicellular organization and regionalization of spheroids^14–16^.

Little is known about the physical characterization of 3D spheroids. Centrifugation, parallel-plate compression, micropipette aspiration and fracture of fused spheroids have been used to quantify tissue rheology^17,18^. However, due to the value of spheroids as *in vitro* tumor models for pre-clinical studies, it is crucial to complete their physical characterization and to determine whether they display, like tumors, growth-induced stress. Moreover, it is important also to assess the impact of spheroid production methods on their physical properties. To address these issues we first adapted the experimental protocol described by Stylianopoulos *et al*. to spheroids to demonstrate that growth-induced stress accumulates during the growth of colon adenocarcinoma-derived spheroids. Then, we characterized the spheroid physical properties and investigated how the different seeding and growth duration conditions affect the spheroid structure and the establishment of residual stress. By coupling experimental data with mathematical modeling and simulations, we found that growth-induced stored stress is lower in spheroids made of a high number of cells and grown for shorter time than in spheroids made of fewer cells and grown for longer time. This difference was associated with modifications of the surface topography and local stiffness. Moreover, the different experimental conditions also significantly influenced spheroid response to irinotecan.

## Results

### Spheroids store growth-induced solid stress

**Figure 1:**
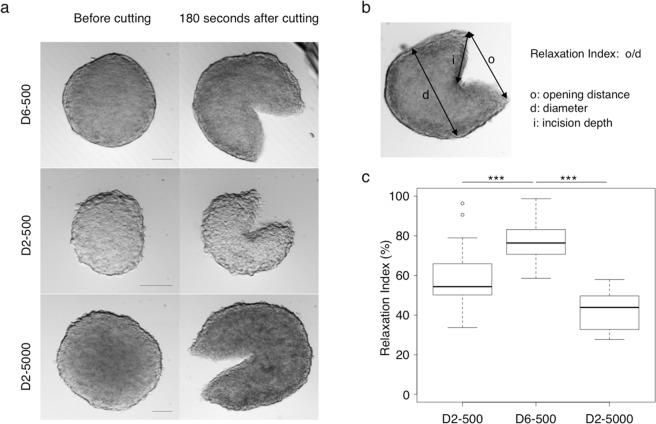
Spheroids accumulate growth-induced solid stress. (**a**) Representative transmitted-light microscopy images of HCT116 cell-derived spheroids prepared starting from 500 cells and grown for 2 or 6 days (D2-500 and D6-500) or from 5,000 cells and grown for 2 days (D2-5000); (left) before cutting and (right) 180 seconds after the incision (approx. 50% of diameter). Scale bar: 100 µm. (**b**) The relaxation index is determined after measuring three parameters: spheroid diameter, incision depth and opening distance. (**c**) Boxplots of the relaxation index of D2-500 (n = 19), D6-500 (n = 29) and D2-5000 (n = 22) spheroids (***p < 0.001). The diameter, incision depths and percentage of incision for each spheroid are shown in Supplementary Fig. S1.

To determine whether tumor spheroids accumulate solid stress, we made a partial incision on each spheroid derived from HCT116 colon carcinoma cells and then monitored the morphological changes by video-microscopy. Such partial cuts resulted in a measurable deformation that involved simultaneously swelling at the center and retraction at the boundary of the spheroid (Fig. 1a and Supplementary Movie 1). In spheroids made starting from 500 cells and grown for 6 days (called thereafter D6-500 spheroids), a clear opening was visible at the site of the cut three minutes after incision.

**Figure 2:**
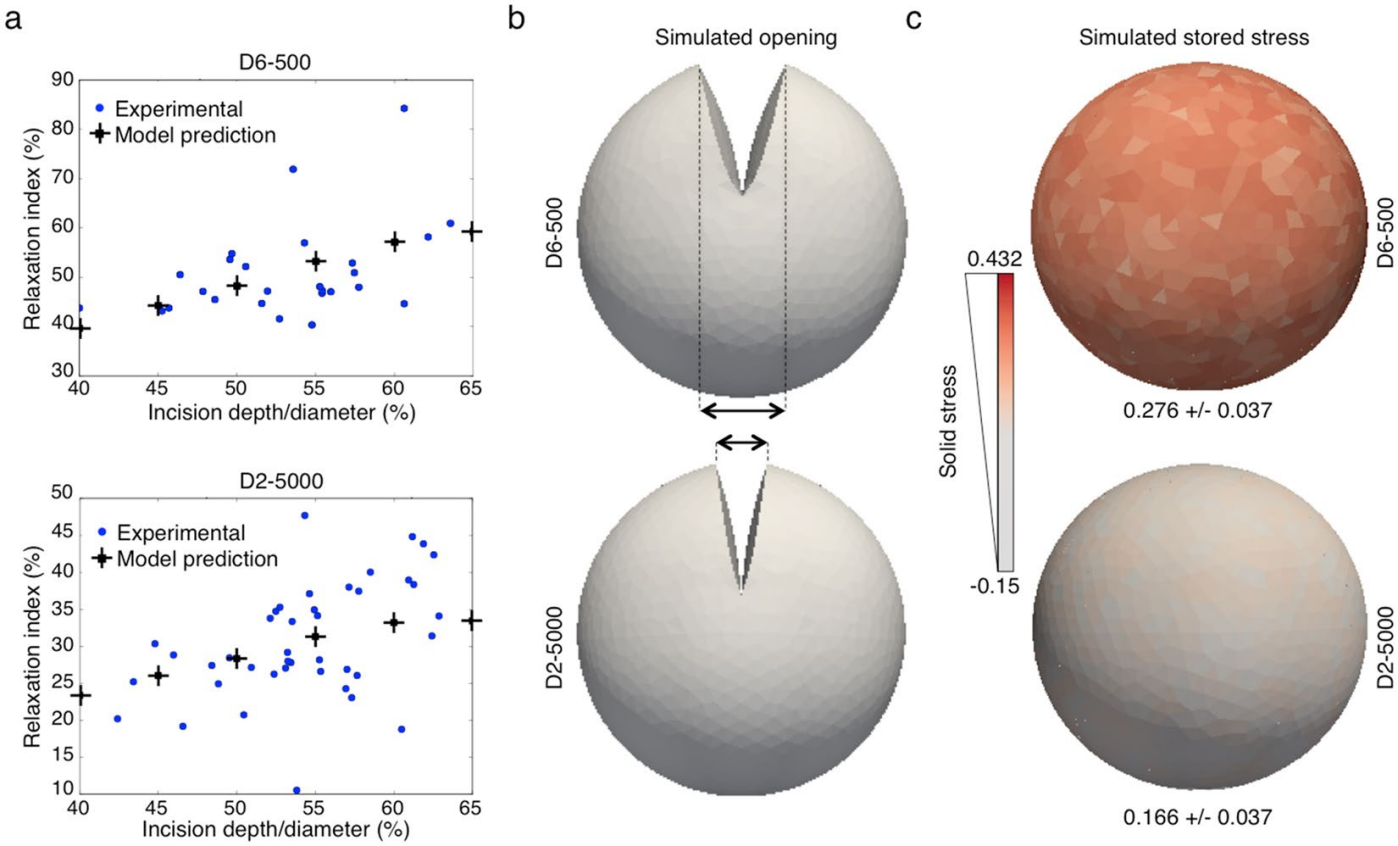
Mathematical modeling of the accumulated growth-induced solid stress in spheroids. A mathematical model was used to simulate the opening of spheroids after the incision and to estimate the growth-induced stress accumulated in D6-500 and D2-5000 spheroids. (**a**) Comparison of the results from the mathematical model predictions (crosses) and the relaxation index calculated from experimental data obtained after spheroid incision at increasing depth (from 40% to 65% of the diameter). (**b**) Simulated images of the opening at 10 seconds after partial incision of D6-500 and D2-5000 spheroids. (**c**) Mathematical modeling of solid stress accumulation. Simulated images of the solid stress accumulated during spheroid growth. Solid stress is expressed in arbitrary units and visualized with a color scale.

We quantified the accumulation of growth-induced solid stress using a relaxation index that corresponds to the extent of the post-incision opening normalized to the spheroid diameter before the partial cut (Fig. 1b,c and Supplementary Fig. S1). In parallel, we used a quantitative mathematical model that proposes a method to estimate the stored stress in a spherical domain on the basis of the opening extent and incision depth (and^19^ see Methods for more details). It assumes that the distribution of the stored stress in a domain at the equilibrium (at rest) results from the relationship between radial and circumferential stored stresses. Combined with a mechanical model of hyperelasticity, it allows simulating the displacement of a spherical domain after an incision. Thus, for a given stored stress, the model predicts the response curve that gives the relaxation index, depending on the incision depth/diameter ratio. To estimate the stored stress predicted by this mathematical model, we made incisions of various depths in D6-500 spheroids and measured the relaxation index for each incision depth. In parallel, we performed a series of simulations for various magnitudes of stored stress, and then compared the obtained response curves with the experimental data (Fig. 2a and Supplementary Fig. S2). We used the stored stress magnitude for which the response curve points belong to the regression line of the experimental points to generate simulated images of spheroid opening that fairly reproduce the experimental observations (Fig. 2b). This analysis allowed determining the stress accumulated within D6-500 spheroids (Fig. 2c). Together, these experimental and mathematical simulation results confirmed the accumulation of solid stress in spheroids at a given growth stage, as reported macroscopically for solid tumors^8^.

### Growth-induced solid stress accumulation depends on spheroid production conditions

Spheroids made starting from 500 cells but grown only for 2 days (D2-500 spheroids) relaxed less after incision compared with D6-500 spheroids (Fig. 1a,c), suggesting that spheroids progressively accumulate solid stress during growth. To exclude that the opening differences between D2-500 and D6-500 spheroids were only the consequence of a volume difference, we compared the response to incision of D6-500 spheroids and of spheroids made from 5,000 cells and grown for 2 days (D2-5000 spheroids). These culture conditions allowed the production of spheroids that reached the same final volume either by proliferation (D6-500 spheroids) or simply by assembling a higher number of cells (D2-5000 spheroids).

Following incision, the opening of D2-5000 spheroids was smaller (Fig. 1a) and relaxation also was significantly lower than in D6-500 spheroids (Fig. 1c). Accordingly, mathematical modeling and simulation indicated that the stored solid stress accumulated during spheroid growth was 1.67-fold higher in D6-500 spheroids (0.276 ± 0.037) than in D2-5000 spheroids (0.166 ± 0.037) (Fig. 2). These data show that growth-induced solid stress is lower in D2-5000 spheroids and that the spheroid production conditions have a very significant impact on this physical property.

### Growth-induced solid stress is associated with difference in spherical shape acquisition depending on the spheroid production conditions

**Figure 3:**
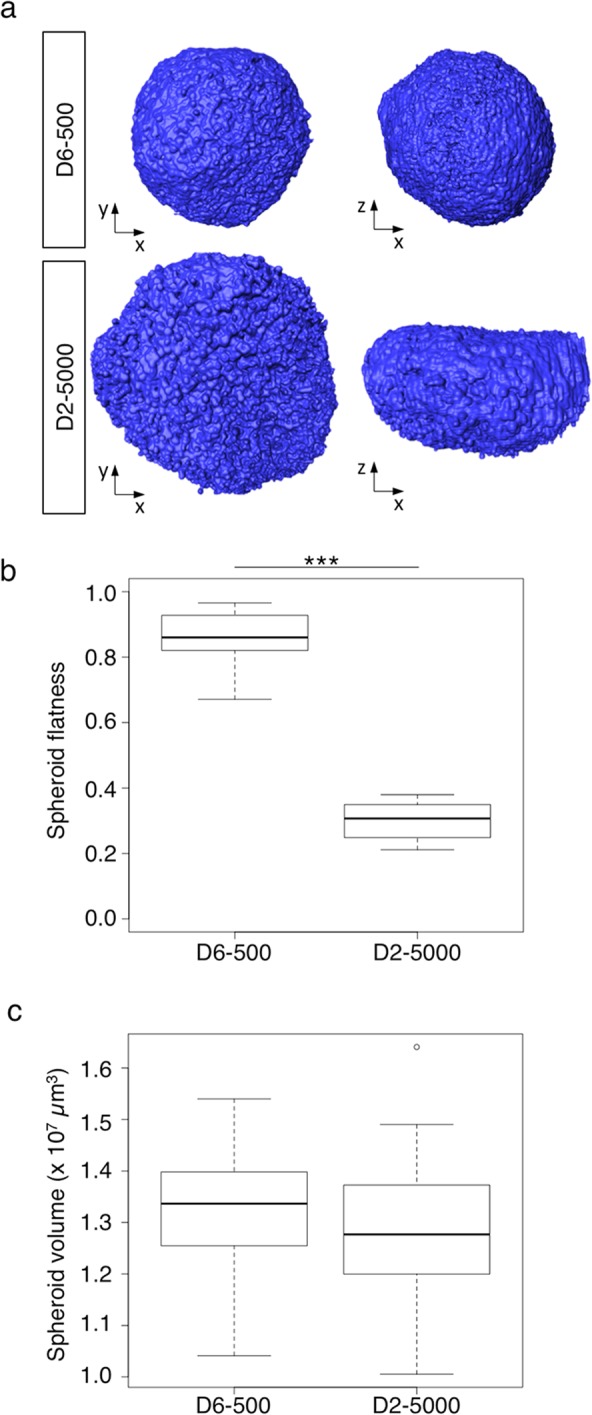
Growth-induced solid stress is associated with differences in spherical shape acquisition depending on the spheroid production conditions. D6-500 and D2-5000 spheroids were fixed, stained with propidium iodide, cleared with a BABB solution and then examined by light sheet microscopy with an optical sectioning of 1 µm step. (**a**) Representative 3D reconstruction and visualization of D6-500 and D2-5000 spheroids using the AMIRA software. Boxplots of the spheroid flatness (**b**) and spheroid volume (**c**) determined using the AMIRA software for D6-500 (n = 31 in 4 independent experiments) and for D2-5000 spheroids (n = 23 in 3 independent experiments); ****p < 0.0001.

We next investigated the relationship between accumulation of solid stress and spheroid multicellular organization. We used light sheet fluorescence microscopy coupled to 3D image analysis to evaluate the volume and flatness of D6-500 and D2-5000 spheroids. To visualize the whole spheroid volume, we used propidium iodide staining and a benzyl alcohol and benzyl benzoate (BABB)-based clearing procedure (see Materials and Methods section) to achieve inner-tissue imaging while preserving the cell morphology and 3D organization^15^. This visualization method (representative images in Fig. 3a) revealed that D6-500 spheroids were quite spherical, while D2-5000 spheroids displayed a flattened shape in the x-y and x-z axes. Importantly, we could not detect these shape differences by using 2D transmitted-light microscopy (see images in Fig. 1a). Determination of the spheroid flatness confirmed this visual observation. While D6-500 spheroids were almost spherical (0.86 ± 0.08), D2-5000 spheroids were extremely flat (0.30 ± 0.05) (Fig. 3b). Conversely, the spheroid volume was comparable in D6-500 and D2-5000 spheroids (Fig. 3c). These observations indicate that morphological changes and spherical shape are associated with the increase in growth-induced solid stress.

### Spheroid surface topology and stiffness are dependent on the production conditions

**Figure 4:**
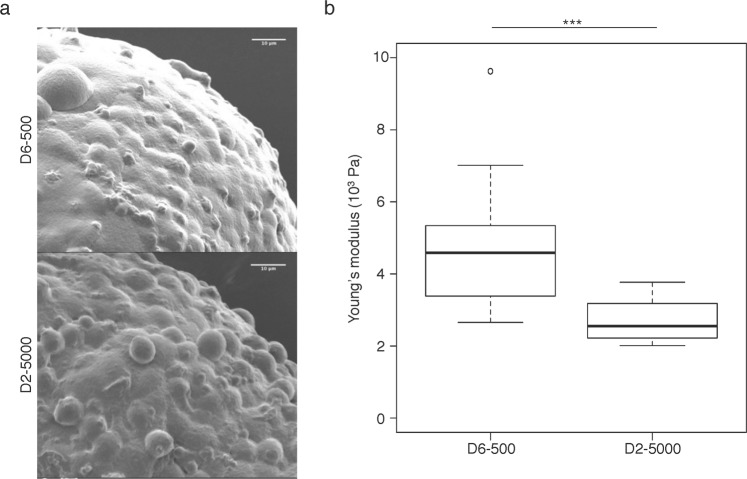
Spheroid surface topology and stiffness are dependent on the production conditions. (**a**) Scanning electronic microscopy images of the surface of D6-500 and D2-5000 spheroids. Scale bar: 10 µm. (**b**) Atomic force microscopy determination of the Young’s modulus in immobilized D6-500 (n = 16 spheroids; total number of indentation curves = 25015) and D2-5000 spheroids (n = 10 spheroids; total number of indentation curves = 10471 curves). Boxplots showing the mean Young’s modulus extracted from the analysis of the curves obtained for each spheroid. Data between the two experimental conditions are significantly different (***p < 0.001).

Analysis of 3D light sheet microscopy images of D6-500 and D2-5000 spheroids suggested that the surface of D2-5000 spheroids was rougher than that of D6-500 spheroids (Fig. 3a). Characterization of the spheroid surface using scanning electronic microscopy showed that the surface of D6-500 spheroids was rather smooth (Fig. 4a and Supplementary Fig. S3). Conversely, the surface of D2-5000 spheroids was more irregular with many cells individually visible at the surface.

Therefore, we investigated the local mechanical properties and measured the spheroid surface stiffness using atomic force microscopy (AFM). The major technical challenge was to immobilize the spheroids for probing the surface with the AFM cantilever. To this aim, we designed and produced 200 µm-high polydimethylsiloxane micro-well arrays with an internal diameter of 450 µm, which corresponds to the spheroid diameter (see Methods and Supplementary Fig. S4). We positioned D6-500 and D2-5000 spheroids in the micro-wells and then analyzed their surface stiffness by measuring the elastic modulus. To take into account the high spheroid size (in relation to the surface analyzed by AFM) and the possible stiffness heterogeneity due to variations between cells and cell-cell junctions, we analyzed different 10 µm^2^ zones in each spheroid to determine an average Young’s modulus (see Methods section and Supplementary Fig. S4). The Young’s modulus values varied between 2 kPa and 10 kPa, in agreement with data obtained in soft tissues^20^. The significantly higher Young’s modulus values for D6-500 spheroids than for D2-5000 spheroids (p < 0.001) (Fig. 4b and Supplementary Fig. S4) indicated that D6-500 spheroids are characterized by higher surface stiffness.

These findings show that the spheroid production conditions have a significant impact on the surface topography and mechanical properties, as indicated by the different surface stiffness.

### The spheroid production conditions modulate the response to irinotecan

**Figure 5:**
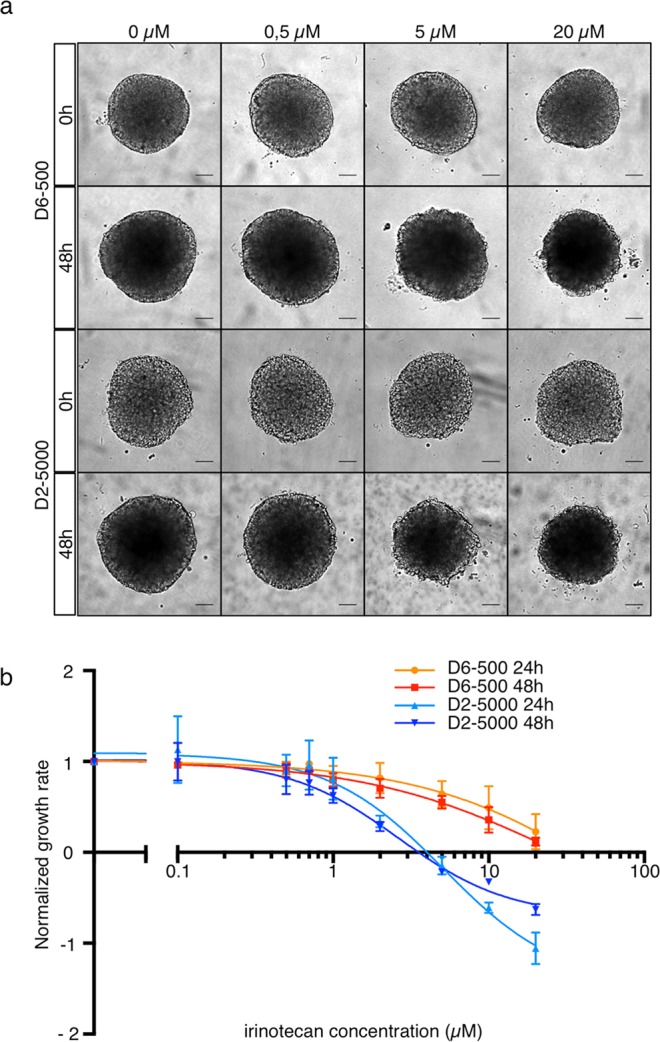
Spheroid production conditions modulate the response to irinotecan. (**a**) Representative transmitted light microscopy images of D6-500 and D2-5000 spheroids incubated or not with increasing concentrations of irinotecan for 48 h, as indicated. Scale bar: 100 µm. (**b**) Comparison of irinotecan growth inhibitory effect after 24 h and 48 h of incubation. For each spheroid, the spheroid projected area was measured by segmentation of the transmitted light microscopy images and the growth rate was calculated. Then, the mean growth rate for the different conditions was normalized to the mean growth rate of untreated control spheroids. Each curve corresponds to the analysis of 14 to 18 spheroids from 3 independent experiments.

As our results showed that the spheroid production conditions influence the accumulated growth-induced solid stress, spheroid shape, surface topography and surface stiffness, we asked whether these effects could modify the spheroid biological properties, such as the response to anticancer drugs. As an example, we incubated D6-500 and D2-5000 spheroids with increasing concentrations of irinotecan, a widely used chemotherapeutic agent, and then measured the spheroid area from transmitted-light images at 24 h and 48 h to determine the growth inhibition. This is a classical strategy to evaluate the activity of anti-proliferative compounds in 3D models. It does not allow considering the difference in shape, but is compatible with longitudinal recording of live spheroids. Exposure to irinotecan for 48 h resulted in growth inhibition in both spheroid types (representative micrographs in Fig. 5a). Quantification of the growth rate (normalized to untreated control) at 24 h and 48 h of incubation showed that at low irinotecan concentration, the growth rates of D6-500 and D2-5000 spheroids were similar. Conversely, at concentrations higher than 5 µM, irinotecan growth inhibitory effect was stronger in D2-5000 spheroids, and this difference increased with higher concentrations (Fig. 5b). These results indicate that D2-5000 spheroids are more responsive to irinotecan that D6-500 spheroids.

## Discussion

The objective of our work was to investigate the physical properties of 3D spheroids and to determine whether, like tumors, they accumulate growth-induced stress^8^. To this aim, we analyzed the physical and biological properties of spheroids of similar volume obtained by seeding different numbers of cancer cells (500 and 5,000) and culturing them for different amounts of time (6 and 2 days).

We first adapted the experimental protocol described by Stylianopoulos *et al*. to demonstrate using micro-dissection experiments and mathematical modeling that 3D spheroids accumulate growth-induced solid stress. We then showed that this growth-induced stored stress is lower in D2-5000 spheroids than in D6-500 spheroids. We also found using light sheet microscopy that the spheroid preparation conditions have a critical impact on the spheroid shape. Indeed, D2-5000 spheroids are not spherical (Fig. 3). This result indicates that accumulation of growth-induced stress is central in the 3D multicellular structuration of a cell aggregate that progressively becomes spherical. In agreement, we also found by scanning electron microscopy that the surface topography is different in D2-5000 and D6-500 spheroids, and by AFM that the surface Young’s modulus is higher in D6-500 spheroids. The Young’s modulus values of our HCT116 cell-derived spheroids (between 2000 and 3500 Pa) are comparable with those (230 and 1250 Pa) reported in a recent publication on the first use of micro-tweezers to measure the stiffness of 3D spheroids derived from breast normal and cancer cell lines^21^. In this previous study, for each cell line, the number of cells to be seeded was chosen to obtain spheroids of the same size, highlighting the fact that adjusting the number of seeded cells is a common experimental habit.

Finally, we showed that accumulation of growth-induced solid stress is associated with a significant modification of the sensitivity to irinotecan. “Young” D2-5000 spheroids were more sensitive than “old” D6-500 spheroids prepared with fewer cells and grown for a longer time. Spheroids are largely used in pre-clinical evaluation because they recapitulate some of the tumor features that are responsible for treatment resistance, including 3D architecture, cell proliferation heterogeneity and cellular physical interactions that constitute potential barriers for drug penetration and distribution^9,14^. In 3D spheroids, drug sensitivity is linked to the cell proliferation status that is largely dependent on their size^9^, and on nutrient and oxygen availability^16^. Here, we show that growth-induced solid stress also plays a central role in the drug response. This could result from modifications in the cell proliferation gradient, or from differences in the amount of extracellular matrix that is involved in the stored stress and represents a barrier for drug diffusion^22^. Moreover, elevated interstitial fluid pressure has been associated with limited drug penetration within tumors, and its level could be influenced by solid stress^8,23^. Indeed, solid stress on spheroid could represent a key parameter in a physical barrier to drug diffusion inside spheroids. Hence, it has to be considered together with pathophysiological gradients when using spheroid models for pre-clinical evaluation.

In recent years, multicellular spheroids have taken a remarkable place in the assessment of new drugs and the interest for these 3D models is currently expanding with applications in basic research and also for pharmacological evaluation. Many production methods have been developed to produce batches of 3D spheroids of homogeneous size in a very reproducible way. However, whatever the production method used, an aspect is never explicitly considered by users: the number of cells used at seeding to produce a spheroid of a required size after a given number of days. Indeed, the only considered parameter is the spheroid size at the beginning of the experiment. For reasons often largely dependent on the users’ habits, or in order to comply with experimental or working schedule constraints, experimenters will adjust both the number of seeded cells and the duration of growth, and sometimes will change these parameters from one experiment to another, in order to obtain spheroids of the required size. As pointed out in a recent review, the parameters for the “optimal spheroid” varies depending on the study objectives, and more often than not, the spheroid production protocol and the reasons for choosing a given method are never discussed^24^.

We consider that our present results should be a warning for the 3D spheroids users’ community. Indeed, the differences in the physical properties/organization and in the response to an anti-tumor agent observed in D2-5000 and D6-500 spheroids should be taken into account by researchers when designing experiments that require 3D spheroids. The parameters of spheroid production must be obligatorily integrated in the assessment and the analysis of results and also for comparison between studies. More generally, tissue engineering is already and will continue to be of tremendous interest for biological research and biomedical applications. Our study demonstrates that special care must be given to the protocol used to produce such tissues to faithfully reproduce the biological and physical properties of a normal tissue.

## Methods

### Cell culture and spheroid generation

HCT116 colorectal carcinoma cells (ATCC CCL-247) were cultured in Dulbecco’s Modified Eagle’s Medium, (DMEM, Gibco # 11574516) containing 10% fetal bovine serum with 100 U/ml penicillin/streptomycin (GIBCO #11548876). Spheroids were prepared as previously described^10,25^. Briefly, 500 cells/well or 5,000 cells/well were distributed in home-made poly-HEMA-coated 96-well round bottom plates (with 100 µL of culture medium. Plates were centrifuged (200 g for 6 min) and then placed in a humidified atmosphere of 5% CO_2_ at 37 °C. This methodology allows obtaining one spheroid per well.

### Spheroid incision experiment and opening analysis

For incision experiments, spheroids were processed one-by-one. After 2 days of growth for spheroids prepared starting from 5,000 cells, and after 2 or 6 days of growth for spheroids derived from 500 cells, one spheroid was transferred to a Petri dish in a drop of 20 µl of PBS using a micropipette. The Petri dish was placed on the stage of a MacroFluo Z16 APO microscope (Leica) fitted with a CoolSNAP ES2 CCD camera (Roper). The incision was done manually along the diameter by using an ophthalmic microscalpel (MicroScalpel Feather®, 15°, Electron Microscopy Sciences #72045-15). During the incision process, the experimenter controlled the positioning of the blade on the spheroid and the incision through the microscope. The incision was rapidly done and bright-field images of spheroids were acquired before, immediately, and at 10 and 180 seconds post-incision to measure the incision length, the opening distance (distance between the two sides of the cut) and the spheroid diameter. These values allowed calculating the relaxation index as follows: (*opening distance*/*spheroid diameter*)*100.

### Analysis of the response to irinotecan treatment

After 6 days (D6-500) or 2 days (D2-5000) of culture, 10 µl of culture medium containing 10 times the required irinotecan (SIGMA #I1406) concentration was added to each well that contained 90 µl of culture medium (n = 6 spheroids/concentration). At the time of irinotecan addition and after 24 h and 48 h of incubation, bright-field images of each live spheroid were acquired with a 5x objective and an inverted microscope (AxioObserver, Zeiss GmbH). Segmentation and shape descriptors were quantified with the ImageJ software^26^. Growth rates at 24 h and 48 h were calculated for each spheroid, relative to the spheroid area at the time of irinotecan addition. For each experiment, the average growth rate at each irinotecan concentration was normalized to the average growth rate of untreated spheroids.

### Light-sheet fluorescence microscopy of optically cleared spheroids

Spheroids were fixed in formalin (Sigma, #HT5014) overnight, then washed twice with PBS and incubated with propidium iodide (10 µg/ml) (Molecular probes #11599296) in the presence of RNase (1 mg/ml) for 30 min (Euromedex #9707). Spheroids were then embedded in 1% low-melting agarose in PBS (Euromedex #1670). Agarose cylinders containing spheroids were collected with a biopsy puncher (8 mm in diameter) and sequentially transferred to 25% (2 h and then overnight), 50% (2 h twice), 70% (2 h twice) and 95% (2 h and then overnight)(v/v) ethanol solutions for complete dehydration. For clearing, samples were transferred in BABB solution (1:2 benzyl alcohol (Alfa Aesar#41218): benzyl benzoate (Sigma#B6630)) for 8 h and then overnight. Images were acquired as previously described^15^. Propidium iodide fluorescence was excited with a 532 nm laser and detected with a 593 nm long-pass filter. The voxel size was 0.645 × 0.645 × 1 μm.

### Global spheroid analysis

The Amira software (Mercury Computer Systems Inc., Chelmsford, MA) was used to create iso-surfaces from the LSFM z-stacks by fluorescence intensity thresholding, and to perform morphometric measurements that were used to determine the spheroid volume and flatness. The same threshold was used for D6-500 and D2-5000 spheroids.

### Atomic force microscopy mechanical measurements and data analysis

PDMS micro-well arrays (see Supplementary Fig. S2) coated with Pluronic-F127 (Sigma, #P2443) to prevent cell adhesion^27^ were fixed to the glass bottom of a Petri dish (FluoroDish WPI#50-823-005, Sarasota, USA) using an oxygen plasma torch (Elveflow, Paris, France). The Petri dish was then filled with 4 ml of culture medium before adding one spheroid in each micro-well using a micropipette. D2-5000 and D6-500 spheroids were placed in different arrays inside the Petri dish that was then carefully placed on the stage of the AFM (Nanowizard II, JPK Instruments, Berlin, Germany) mounted on an inverted microscope (Zeiss, Germany). This setup is fitted with a Petri dish heater (JPK) set to 37 °C and under a flow of 5% CO_2_ to maintain the physiological conditions during the measurements. The deflection sensitivity of the MLCT cantilever (Bruker, Germany) was obtained by calculating the force-distance curve on the glass bottom of the Petri dish 30 min after placing the cantilever in the liquid medium to allow the cantilever to reach the thermal equilibrium. The thermal tune method available for the AFM system was used to calibrate the spring constant of the cantilever far from the surface. The calibrated spring constants were 0.13–0.16 N/m. The “force mapping” mode was used to calculate the force-distance curves over 100 µm^2^ zones on top of each spheroid. The maximum applied force was kept at 1 nN during force mapping and for the initial approach. The JPK Data analysis software was used to extract the apparent Young’s modulus form each force curve. Each approach curve was first smoothed using a 3 pixels Gaussian filter, and then force vs. vertical deflection curves were converted into force vs. tip separation curves before fitting the Conical Hertz Sneddon model:

$$F=\frac{2}{\pi }\frac{E}{(1-{\nu }^{2})}\,\tan \,\alpha \,{\delta }^{2}$$

where F is the Force, E is the apparent Young’s modulus, α is the cantilever tip half angle, 17.5° for MLCT probes, ν is the Poisson ratio set to 0.5 in liquid and δ is the indentation depth.

### Estimation of the stored growth induced-stress using mathematical modeling

The mathematical model that describes the stored growth-induced stress and its effect on spheroid opening upon incision has been described in^19^ and we recall here the key points. The elastic model is hyperelasticity, and we used a Ciarlet-Geymonat material with a Poisson coefficient ν = 0.46 and Ciarlet-Geymonat parameter a′ = 0.4. The stored stress distribution is assumed to have a spherical symmetry. It was proved that radial and tangential stored stresses are linked, and they are defined by a magnitude and a radial profile. The radial profile is assumed to follow a power law with exponent n. To estimate the value of the total radial stored stress and its standard deviation, series of simulations were performed for various magnitudes of stored stress (see Supplementary Fig. S2). To compare the model response and the experimental data (opening/diameter versus incision depth/diameter), for each value γ of the magnitude of stored stress, the number of data points below the response curve gives the fraction of data points that correspond to a stored stress below the value γ. This allows sampling the cumulative distribution function (cdf) of the stored stress magnitude. Fitting the cdf of a Gaussian distribution to these points provides the values of stored stress and the standard deviation (see Supplementary Fig. S2).

### Statistical analysis

Graphs and data were analyzed with GraphPad Prism version 6.00 (GraphPad software, La Jolla California USA, www.graphpad.com) and R (R Core Team 2017, https://www.R-project.org/). The non-parametric Mann-Whitney test was used to compare data from different conditions. Stacks were analyzed using the Amira, Imaris and Fiji^28^ software programs.

## Supplementary information

Supplementary material
