# Supplementary material for: Characterization of the physical properties of tumor-derived spheroids reveals critical insights for pre-clinical studies
Source: Sci Rep. 2019 Apr 29;9:6597. doi: 10.1038/s41598-019-43090-0 (PMC6488646; doi:10.1038/s41598-019-43090-0)
Supplement: Supplementary file 1 — Supplementary material [file 41598_2019_43090_MOESM1_ESM.docx]

**Characterization of the physical properties of tumour-derived spheroids reveals critical insights for pre-clinical studies.**

Ludivine GUILLAUME^1^, Lise RIGAL^1^, Jérôme FEHRENBACH^1,2^, Childérick SEVERAC^1^, Bernard DUCOMMUN^1,3^, and Valérie LOBJOIS^1*^

**SUPPLEMENTARY DATA**

**Supplementary Movie 1:**

**Spheroids accumulate growth-induced solid stress**

Representative images of the spheroid opening after partial incision of HCT116 cell-derived spheroids prepared from 500 cells and grown for 6 days (left) and from 5,000 cells grown for 2 days (right).

**Supplementary Figure S1:**

**Measuring the diameter, incision depth and incision percentage of D2-500, D6-500 and D2-5000 spheroids.**

Scatter plots of the values for the diameter, the incision depth and the incision depth/diameter ratio (giving the incision percentage) for the D2-500, D6-500 and D2-5000 spheroids included in the results shown in Figure 1. For each condition, the mean ± SD is shown in blue. The incision percentage is quite similar for the three conditions with a coefficient of variation of 11.6%, 11% and 10.3% for D2-500, D6-500 and D2-5000 spheroids, respectively.

**Supplementary Figure S2:**

**Determination of the value of the stored stress and the standard deviation.**

(a) The experimental points for one experiment (D6-500) are plotted in blue. The black lines represent the model prediction of the spheroid opening relative to the incision depth. Each value γ of the magnitude of stored stress allows computing one black line, which is the response of the model for the value γ. For the sake of readability, only a few response curves are shown. (b) The experimental sampled cdf points (in blue) and the fitted Gaussian cdf (in black). The fit was performed by minimizing the total least square distance between the points and the curve.

**Supplementary Figure S3:**

**Spheroid surface topology**

Representative scanning electronic microscopy images of the surface of spheroids produced with 5,000 cells and grown for 2 days (D2-5000) or from 500 cells and grown for 6 days (D6-500). Scale bar: 10 µm.

**
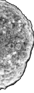
Supplementary Figure S4:**

a

**
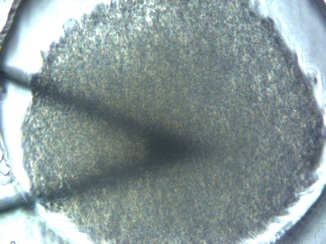
**

*200 µm*

*450 µm*

*Side view*

*Bottom view with*

*cantilever above*

*Array of PDMS microdevices*

**
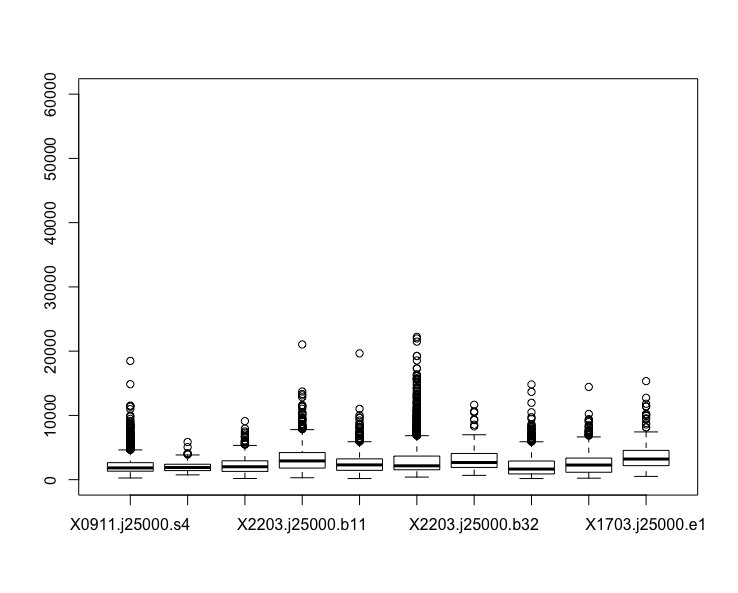

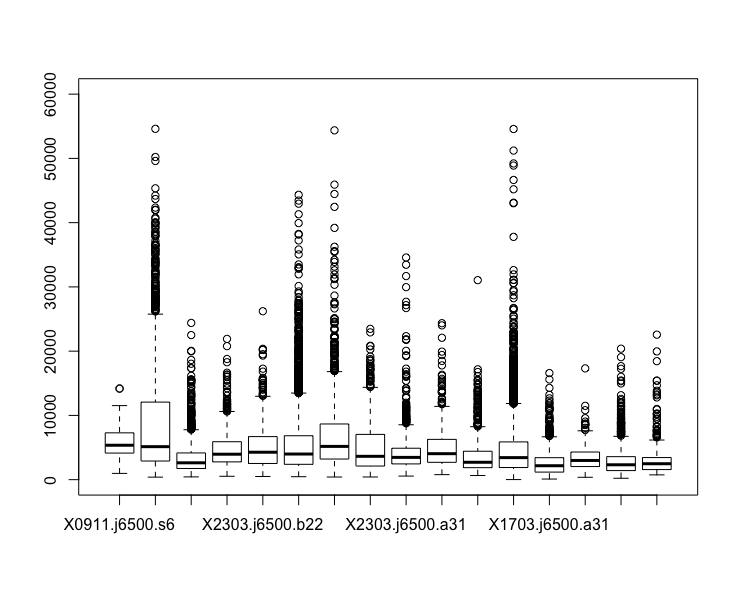
**

b

D6-500

D2-5000

60

60

50

50

40

40

30

30

Young’s modulus (10^3^ Pa)

20

20

10

10

10

9

8

7

6

5

4

1

11

12

1414

0

0

2

3

13

15

16

1

2

3

4

5

6

7

8

9

10

n= 10 spheroids

10471 indentations curves

n= 16 spheroids

25015 indentations curves

**Experimental strategy to perform atomic force microscopy analysis of the spheroid surface stiffness and raw results**

(a) Array of PDMS micro-devices constituted of 16 micro-wells (450 µM internal diameter and 200 µM high). Schematic representation of the positioning and immobilization of a spheroid in a PDMS micro-well to allow probing with the AFM cantilever (seen on the left in the inverted microscopy image).

(b) Boxplots of the Young’s modulus values calculated for each indentation curve obtained for each D6-500 and D2-5000 spheroid, respectively.
